# Genomic insights into the evolution, pathogenicity, and extensively drug-resistance of emerging pathogens *Kluyvera* and *Phytobacter*

**DOI:** 10.3389/fcimb.2024.1376289

**Published:** 2024-03-21

**Authors:** Zhenzhou Huang, Guozhong Zhang, Zhibei Zheng, Xiuqin Lou, Feifei Cao, Lingyi Zeng, Duochun Wang, Keyi Yu, Jun Li

**Affiliations:** ^1^ Microbiology Laboratory, Hangzhou Center for Disease Control and Prevention, Hangzhou, Zhejiang, China; ^2^ National Institute for Communicable Disease Control and Prevention, Chinese Center for Disease Control and Prevention, Beijing, China

**Keywords:** *Kluyvera*, *Phytobacter*, evolution, pathogenicity, drug-resistance

## Abstract

**Introduction:**

*Kluyvera* is a Gram-negative, flagellated, motile bacillus within the *Enterobacteriaceae*. The case reports of clinical infections shed light on the importance of this organism as an emerging opportunistic pathogen. The genus *Phytobacter*, which often be misidentified with *Kluyvera*, is also an important clinically relevant member of the *Enterobacteriaceae*. However, the identification of *Kluyvera* and *Phytobacter* is problematic, and their phylogenetic relationship remains unclear.

**Methods:**

Here, 81 strains of *Kluyvera* and 16 strains of *Phytobacter* were collected. A series of comparative genomics approaches were applied to the phylogenetic relationship reconstruction, virulence related genes profiles description, and antibiotic resistance genes prediction.

**Results:**

Using average nucleotide identity (ANI) and *in silico* DNA-DNA hybridization (*is*DDH), we offered reliable species designations of 97 strains, in which 40 (41.24%) strains were incorrectly labeled. A new *Phytobacter* genomospecies-1 were defined. *Phytobacter* and *Kluyvera* show great genome plasticity and inclusiveness, which may be related to their diverse ecological niches. An intergenomic distances threshold of 0.15875 was used for taxonomy reassignments at the phylogenomic-group level. Further principal coordinates analysis (PCoA) revealed 11 core genes of *Kluyvera* (*pelX, mdtL, bglC, pcak-1, uhpB, ddpA-2, pdxY, oppD-1, cptA, yidZ, csbX*) that could be served as potential identification targets. Meanwhile, the *Phytobacter* specific virulence genes *clbS, csgA-C, fliS, hsiB1_vipA and hsiC1_vipB*, were found to differentiate from *Kluyvera*. We concluded that the evolution rate of *Kluyvera* was 5.25E-6, approximately three times higher than that of *Phytobacter*. Additionally, the co-existence of ESBLs and carbapenem resistance genes were present in approximately 40% strains, suggesting the potential development of extensively drug-resistant or even fully drug-resistant strains.

**Discussion:**

This work provided a better understanding of the differences between closely related species *Kluyvera* and *Phytobacter*. Their genomes exhibited great genome plasticity and inclusiveness. They not only possess a potential pathogenicity threat, but also a risk of multi-drug resistance. The emerging pathogens *Kluyvera* and *Phytobacter* warrant close attention.

## Introduction

*Kluyvera* is a Gram-negative, flagellated, motile bacillus within the *Enterobacteriaceae*. Members of this genus were phenotypically identical to *Escherichia* including the ability to use citrate, malonate, decarboxylate lysine, and ornithine and to produce large quantities of α-ketoglutaric acid during the glucose fermentation (Sarria et al., 2001). It can be isolated from various clinical and environmental sources such as freshwater, seawater, sewage, soil and rhizosphere (Sarria et al., 2001; Gessew et al., 2022; Paschoal et al., 2017; Li Y. et al., 2019; Yim et al., 2009). Since its redefinition in 1981 (Farmer et al., 1981), *Kluyvera* has been recognized as an important pathogen in human disease (Sarria et al., 2001), with reported infections in the gastrointestinal or urinary tract, the soft tissues, and cases of bacteremia and systemic infections involving multiple organs (Bolat et al., 2013; Erbin et al., 2020; Sierra-Madero et al., 1990; Sezer et al., 1996). *Kluyvera* also serves as a reservoir and communicator of the *bla*_CTX-Ms_ genes, which are responsible for the production of Extended-Spectrum Beta-Lactamases (ESBLs). The chromosomal *bla*_CTX-Ms_ genes from *Kluyvera* have been shown to be the progenitors of the plasmid-encoded *bla*_CTX-Ms_ (Cantón et al., 2008).

In recent years, the taxonomy of *Kluyvera* has become increasingly complex with the identification of new genomospecies. Currently, there are five recognized species (*K. ascorbata*, *K. cryocrescens*, *K. georgiana*, *K. intermedia*, *K. sichuanensis*). Liu et al. identified four *Kluyvera* genomospecies according to the available sequences in Genbank (Liu et al., 2020). Rodríguez et al. proposed a new genomospecies-5 (Rodríguez et al., 2021). The classification of some genome sequences in NCBI has shown inconsistency with *Phytobacter* (Rodríguez et al., 2021; Smits et al., 2022), further emphasizing the need for clarification of genus taxonomy reassignments.

The taxonomic status of *Phytobacter* has also undergone a complex process. Originally described in 2008, *Phytobacter* and its type species *Phytobacter diazotrophicus* were isolated as endophytic nitrogen-fixing bacteria from wild rice in China (Zhang et al., 2008). However, subsequent research revealed that clinical isolates from a multi-state sepsis outbreak in Brazil also belonged to *Phytobacter diazotrophicus* (Pillonetto et al., 2018a). Moreover, a new *Phytobacter* species, *Phytobacter ursingii*, was identified among American clinical strains (Pillonetto et al., 2018a). *Phytobacter* has been found to occupy various ecological niches and can adapt to clinical environments, potentially becoming a multidrug-resistant microorganism (Smits et al., 2022; Sekizuka et al., 2018; Pillonetto et al., 2018b; Zhang et al., 2020).

Based on previous investigations, *Phytobacter* may have similar clinical relevance to *Kluyvera* (Smits et al., 2022). Despite their clinical significance, the genomic characteristics, virulence and resistance signature of *Kluyvera* and *Phytobacter* remain poorly understood. In this study, we conducted a comprehensive analysis of 97 high-quality draft genome sequences, including four newly sequenced strains, to provide new insights into the species status, genetic relatedness, potential pathogenicity and resistance genes diversity of these genera.

## Materials and methods

### Curation of *Kluyvera, Phytobacter* genomes available in GenBank

Ninety-three genomes belonging to the genus *Kluyvera* (originally labeled: n=77) and *Phytobacter* (originally labeled: n=16) were downloaded from the NCBI FTP server (ftp.ncbi.nih.gov) and SRA database (https://www.ncbi.nlm.nih.gov/sra). Only genomes with less than 200 contigs were selected for analysis. Additional four newly sequenced strains isolated from China (deposited in the Centre for Human Pathogenic Culture Collection, China CDC) were included in the study (GenBank accession: PRJNA899585). These strains were initially identified by 16S rRNA, API 20E and API 20NE characterization methods. Detailed information of selected strains was listed in **Supplementary Table 1**. Overall, a total of 93 publicly available genomes and four newly sequenced strains were used for the analysis.

### Genome sequencing and assembly

The strains were cultured on LB-1% NaCl agar at a temperature of 37°C. The genomic DNA from the four isolated strains was performed using the Wizard^@^ Genomic DNA Extraction Kit (Madison, WI, Promega, USA) following the manufacturer’s instructions. Subsequently, the extracted DNA samples were subjected to 250-bp paired-end whole genome sequencing with 150× coverage using the HiSeq sequencer (Illumina HiSeq2000, San Diego, CA, USA). And the resulting reads were *de novo* assembled into contigs using SPAdes v3.13.0 (Bankevich et al., 2012).

### Phylogenomic analysis

Average nucleotide identity (ANI) calculations (Richter and Rosselló-Móra, 2009) and the genome-to-genome blast distance phylogeny (GBDP) algorithm were used for the phylogenomic analysis, which replicates the DNA-DNA hybridization digitally for species delineation (Meier-Kolthoff et al., 2013). Minimal cutoff points of 70% *in silico* DNA-DNA hybridization (*is*DDH) and 95% ANI values were considered to represent species delineation. Intergenomic distances among the 97 identified strains were calculated by using the genome-to-genome distance calculator web service (GGDC, http://ggdc.dsmz.de/) (Meier-Kolthoff et al., 2013). These distances were then transformed into a matrix and imported into FastMe v2.0 (Lefort et al., 2015) to build the neighbor-joining (NJ) phylogenomic tree. *Aeromonas hydrophila* ATCC 7966^T^ was served as the outgroup. To assess strain identification at the genus or species level, the online TYGS platform (https://tygs.dsmz.de/user_requests/new) was utilized (Meier-Kolthoff and Göker, 2019). Clustering of intergenomic distances was examined using the OPTSIL v1.5 (Garrido-Sanz et al., 2020), which creates a non-hierarchical clustering using distance threshold *T* and a specific *F* value. *F* values ranging from 0 to 1 indicate the fraction of links required for cluster fusion. *F* value of 0.5 represents average-linkage clustering. In this study, the distance threshold (*T*) values ranging from 0 to 0.3 were used with a step size of 0.0005. The best *T* and *F* for both, phylogenomic-group level and species level were chosen based on the highest modified Rand Index (MRI) score.

### Core- and pan- analysis

A local database containing 97 selected strains sequences was created for further analysis. The genomes were annotated using Prokka v1.12 (Seemann, 2014), which generated *.gff output files for each strain. The Roary pan-genome pipeline was then used with an identity cutoff of ≥95% (Page et al., 2015). Prodigal v2.6.3 was used for coding sequence prediction (Hyatt et al., 2010). To extract the core genes from all the selected strains, CD-HIT v4.6.6 was employed to create a non-redundant set of homologous genes (Fu et al., 2012). Next, BLAST+ was used to search for homologous genes in this non-redundant set with a minimum identity ≥90% and length coverage ≥60%. The identified core genes were aligned, and Gubbins (http://github.com/sanger-pathogens/gubbins) was used as a recombination-removal tool to reorganize the core genome. Phylogenetic trees were constructed using PhyML v3.1 by maximum-likelihood method with 1000 bootstrap replications (Guindon et al., 2010). Population structure was defined using FastBaps (https://github.com/gtonkinhill/fastbaps) by a fast hierarchical Bayesian analysis (Tonkin-Hill et al., 2019). Snippy v4.3.6 was used to extract core SNPs, which were then used to build a maximum-likelihood phylogenetic tree. Principal coordinate analysis (PCoA) based on Bray-Curtis distance and Jaccard distance was performed to assess the comprehensive composition of the accessory genes of these strains. Principal component analysis (PCA) was performed with prcomp function in R v3.6.1 based on the presence of common (5–95% prevalence) accessory genes.

### Evolutionary analysis of *Kluyvera* and *Phytobacter*

We conducted evolutionary analysis on the core genes of *Kluyvera* and *Phytobacter*, and estimated the evolutionary origin time for each genus using BEAST v1.10.4 (Hill and Baele, 2019). The molecular clock was calibrated using the isolation date (by year of collection) for each strain. A GTR+Γ substitution model and uncorrelated log-normal relaxed clock were used in our analysis. The tree prior’s parameters were specified based on a normal distribution. For *Kluyvera*, we performed a Markov chain Monte Carlo (MCMC) of 1×10^-8^ iterations and sampled every 20,000 iterations to assure independent convergence of the chains. Similarly, for *Phytobacter*, we ran three independent Markov chain Monte Carlo of 1×10^-8^ iterations and sampled every 20,000 iterations. Convergence of the MCMC chains was assessed using Tracer v1.6.0, ensuring that all relevant parameters reached an effective sample size >200.

### Pan virulence-related genes analysis

In this study, we analyzed the virulence-related gene profiles of 97 strains. The virulence-related genes were annotated according to the Virulence Factors Database (VFDB, http://www.mgc.ac.cn/VFs/). The protein sequences of selected strains were searched against the VFDB database using BLASTp with the parameters of E-value of 1e-5, identity ≥60% and coverage ≥70%. The pan virulence-related genes analyzed in this study included both core virulence-related genes, which were present in all strains, and accessory virulence-related genes (Li Z. et al., 2019), which were only present in some strains.

### Identification of antibiotic resistance genes

Antibiotic resistance genes were predicted using Comprehensive Antibiotic Research Database (CARD) (http://arpcard.mcmaster.ca), with the parameters of BLAST+ were E-value of 1e-5, identity ≥80% and coverage ≥80%.

## Results

### Strains re-identification and genomic characteristics analysis

**Figure 1:**
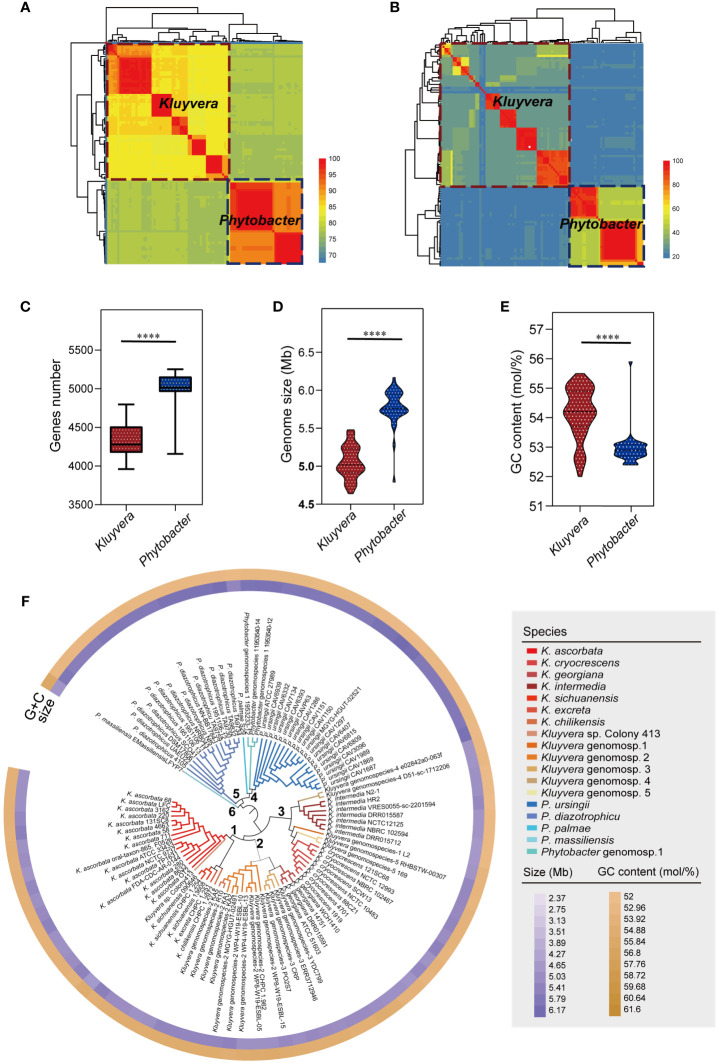
Strains re-identification and genomic characteristics analysis. **(A)** Species-level clusters with an ANI threshold at 95%. *Aeromonas hydrophila* ATCC 7966^T^ served as an outgroup. **(B)** Species-level clusters with a *is*DDH threshold at 70%. *A. hydrophila* ATCC 7966^T^ served as an outgroup. **(C)** Comparison of the genes number of *Kluyvera* and *Phytobacter*. **(D)** Comparison of the genome size of *Kluyvera* and *Phytobacter*. **(E)** Comparison of the GC content (mol/%) of *Kluyvera* and *Phytobacter*. Two groups with significant differences were marked with **** (*P* < 0.0001). **(F)** GBDP-based phylogeny of *Kluyvera* and *Phytobacter* genomes.

Based on ANI and *is*DDH analysis, the multiple inconsistencies regarding the current assignment to species were observed (**Figures 1A, B**). A total of 40 (41.24%) strains were incorrectly labeled. Among them, 21 strains were misidentified within two genera and 19 strains were misidentified within the single genus. Finally, 60 strains belonging to the genus *Kluyvera* (*K. ascorbata*: n=16; *K. cryocrescens*: n=8; *K. excreta*: n=1; *K. georgiana*: n=4; *K. intermedia*: n=7; *K. sichuanensis*: n=3; *K. chilikensis*: n=1; *Kluyvera* genomospecies-1: n=1; *Kluyvera* genomospecies-2: n=10; *Kluyvera* genomospecies-3: n=4; *Kluyvera* genomospecies-4: n=2; *Kluyvera* genomospecies-5: n=2; *Kluyvera* sp.: n=1) and 37 strains belonging to the genus *Phytobacter* (*P. diazotrophicus*: n=14; *P. ursingii*: n=18; *P. palmae*: n=1; *P. massiliensis*: n=1; *Phytobacter* genomospecies-1: n=3) were involved in the taxonomy reassignments. Detailed information was listed in the **Supplementary Table 1**.

Further analysis revealed significant differences in the genome size, GC content, and gene count between *Kluyvera* and *Phytobacter* strains (*P* < 0.05, **Figures 1C–E**). The genome sizes ranged from 2.37 to 6.17 Mb (*Kluyvera*: 2.37-5.48 Mb; *Phytobacter*: 4.79-6.17 Mb), while GC content ranged from 52 to 55.9% (*Kluyvera*: 52-55.5 mol%; *Phytobacter*: 52.4-55.9 mol%). Genes count ranged from 3961 to 5253 (*Kluyvera*: 3961-4797; *Phytobacter*: 4157-5253).

### Phylogenomic analysis of *Kluyvera* and *Phytobacter*

A phylogenomic tree was constructed based on the intergenomic distances (**Figure 1F**) using GBDP algorithm (Meier-Kolthoff et al., 2013). At the phylogenomic-group level, the distance threshold of 0.15875 was used for single-linkage clustering (*F*=0), resulting in complete consistency with the re-identification (i.e., MRI=0). The tree revealed six distinct clusters. Cluster 1 comprised *K. ascorbata*, *K. sichuanensis*, *Kluyvera excreta*, *Kluyvera chilikensis*. Cluster 2 included *Kluyvera* genomospecies-2, *Kluyvera georgiana*, *Kluyvera* genomospecies-3, with *Kluyvera georgiana* and *Kluyvera* genomospecies-3 showing a closer kinship. Cluster 3 encompassed several species with *K. intermedia* and *Kluyvera* genomospecies-4 clustered together, while *Kluyvera* genomospecies-1, *Kluyvera* genomospecies-5, and *K. cryocrescens* clustered together. Cluster 4 consisted of *P. palma*, *Phytobacter* genomospecies-1, and *Phytobacter ursingii*, while *P. diazotrophicu* formed its own cluster, Cluster 5. *P. massiliensis* showed a clear separation from other *Phytobacter* strains and formed Cluster 6. A distance threshold of *T* = 0.036, equivalent to 70% of *is*DDH, was used for taxonomy reassignments at the species level. This threshold resulted in 18 species clusters that showed complete consistency with the reference partition (i.e., MRI=0) when using single-lineage clustering (*F*=0). These findings validated the species-level re-identification in this study. Among the species clusters, 13 belong to the *Kluyvera* subgroup, while five belong to *Phytobacter*.

### Genetic and functional difference among two genera

**Figure 2:**
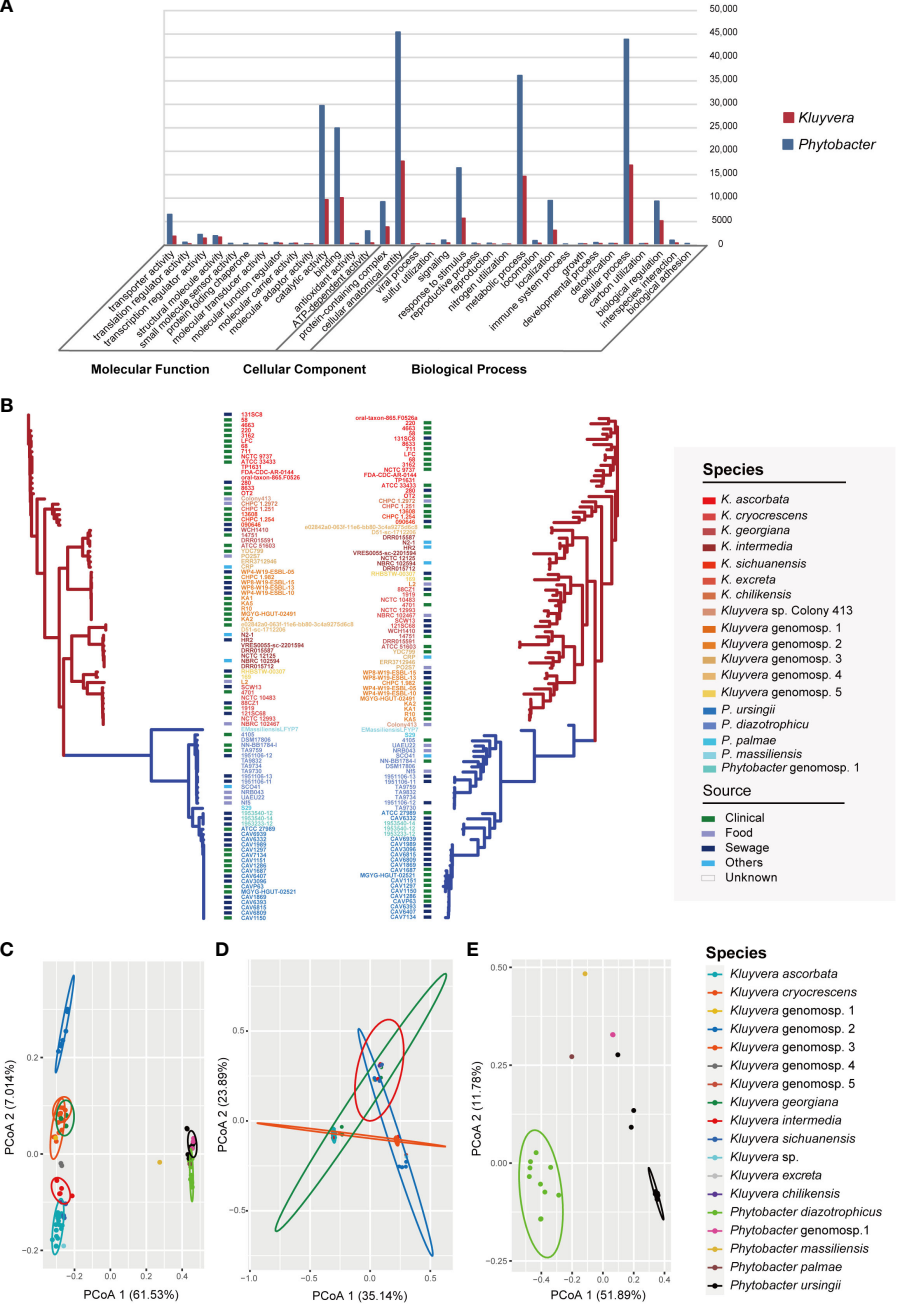
Pan-genome analysis of *Kluyvera* and *Phytobacter* strains. **(A)** Functions and gene numbers of core genes of *Kluyvera* and *Phytobacter.* **(B)** Phylogenetic tree of core genome sequences (left) and accessory genes (right) using the maximum likelihood method. **(C)** PCoA analysis of accessory genes of 97 strains. **(D)** PCoA analysis of 60 *Kluyvera* stains. **(E)** PCoA analysis of 37 *Phytobacter* stains.

A total of 19,150 pan genes and 1201 core genes were identified among the 97 strains. For a single genus, the core genes (99% ≤ strains ≤ 100%) number of 60 *Kluyvera* strains was 1164, and that of 37 *Phytobacter* strains was 1886. The functions and gene numbers of core genes of *Kluyvera* and *Phytobacter* are depicted in **Figure 2A**. A maximum-likelihood phylogenetic tree based on core genes SNPs was constructed (**Figure 2B**), showing that the clustering pattern based on core genes SNPs of 97 strains closely resembled that based on whole-genome sequences. The two genera, *Kluyvera* and *Phytobacter*, were clearly separated, and the relative positions of the species remained unchanged. Notably, the clade of *K. ascorbata* showed deeper evolutionary roots, suggesting indicating a more ancient lineage. However, in terms of the phylogenetic tree topology, there were some differences in the accessory genome-based phylogenetic tree compared to the phylogenetic tree based on the core genome (**Figure 2B**). The branch length of the accessory genome-based phylogenetic tree was longer than that of the core genome, reflecting a longer genetic distance. In accessory genome-based phylogenetic tree, strains of the same species, those with similar niches were almost all clustered together, showing a more similar accessory genes composition. Besides, the relative position of some strains has changed. For example, the strain Colony413 was closed with *K. ascorbata* in the core genome-based phylogenetic tree, while it was closed with *Kluyvera* genomospecies-2 in the accessory genome-based phylogenetic tree (**Figure 2B**).

The results of principal coordinates analysis (PCoA) exhibited clear separation among the strains based on the comprehensive composition of accessory genes (**Figures 2C–E**). *Kluyvera* genomospecies-2 was found to harbor sufficient species-specific genes to differentiate it from other species. Meanwhile, principal component analysis (PCA) was also performed based on the accessory genome, with the first and second principal components explaining 38.834% of the variation. Genus-specific genes of *Kluyvera* were identified, including *pelX* (Pectate disaccharide-lyase), *mdtL* (Multidrug resistance protein MdtL), *bglC* (Aryl-phospho-beta-D-glucosidase BglC), *pcaK_1* (4-hydroxybenzoate transporter PcaK), *uhpB* (Signal transduction histidine-protein kinase/phosphatase UhpB), *ddpa_2* (putative D,D-dipeptide-binding periplasmic protein DdpA), *pdxY* (Pyridoxal kinase PdxY), *oppD_1* (Oligopeptide transport ATP-binding protein OppD), *cptA* (Phosphoethanolamine transferase CptA), *yidZ* (HTH-type transcriptional regulator YidZ), and *csbX* (Alpha-ketoglutarate permease).

### Divergence evolutionary analysis among two genera

**Figure 3:**
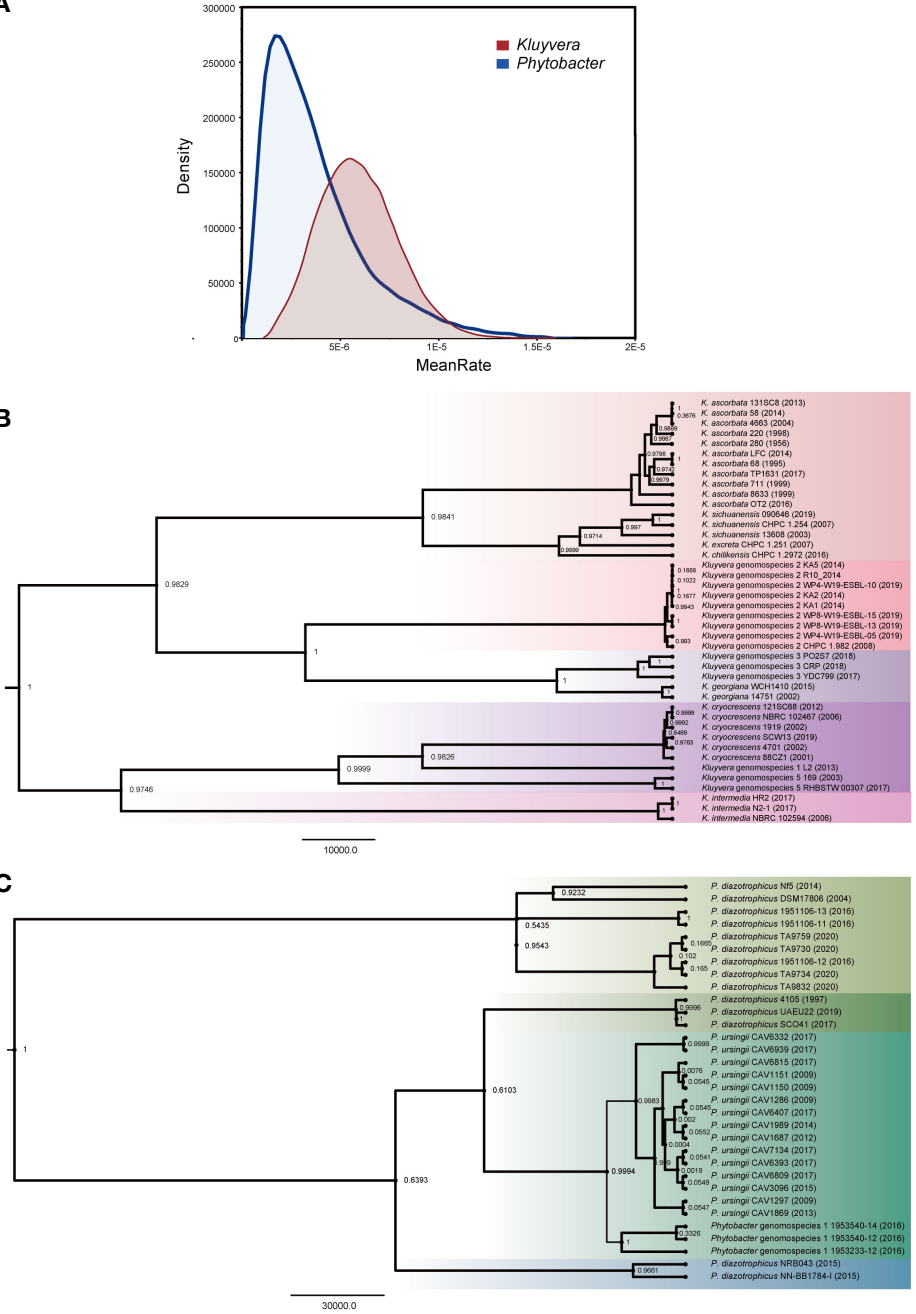
Phylogenetic analysis based on the core genes of *Kluyvera* and *Phytobacter*. The value on the node represents posterior. **(A)** The curve of evolutionary rate. **(B)** The Maximum Clade Credibility Tree (MCC Tree) of *Kluyvera*. **(C)** The Maximum Clade Credibility Tree (MCC Tree) of *Phytobacter*.

The isolation year of the strains was utilized to infer the location of ancestral nodes on the Bayesian family tree. Different colored branches on the tree represented different bacterial species, with the abscissa corresponding to the predicted divergence time (**Figures 3B, C**). The average evolution rate of *Kluyvera* was 5.25E-6, approximately three times higher than that of *Phytobacter*, which had an average evolution rate of 1.85E-6 (**Figure 3A**). The family tree of *Kluyvera* showed two main groups. Group A included *K. ascorbata*, *K. sichuanensis*, *K. excreta*, *K. chilikensis*, *Kluyvera* genomospecies-2, *Kluyvera* genomospecies-3, *K. geogiana*. And group B comprised *K. intermedia*, *K. cryocrescens*, *Kluyvera* genomospecies-1, and *Kluyvera* genomospecies-5. Regarding *Phytobacter*, group A solely consisted of *P. diazotrophicus*, while group B included *P. diazotrophicus*, *P. ursingii*, and *Phytobacter* genomospecies-1.

### Pan virulence genes analysis of *Kluyvera* and *Phytobacter*

**Figure 4:**
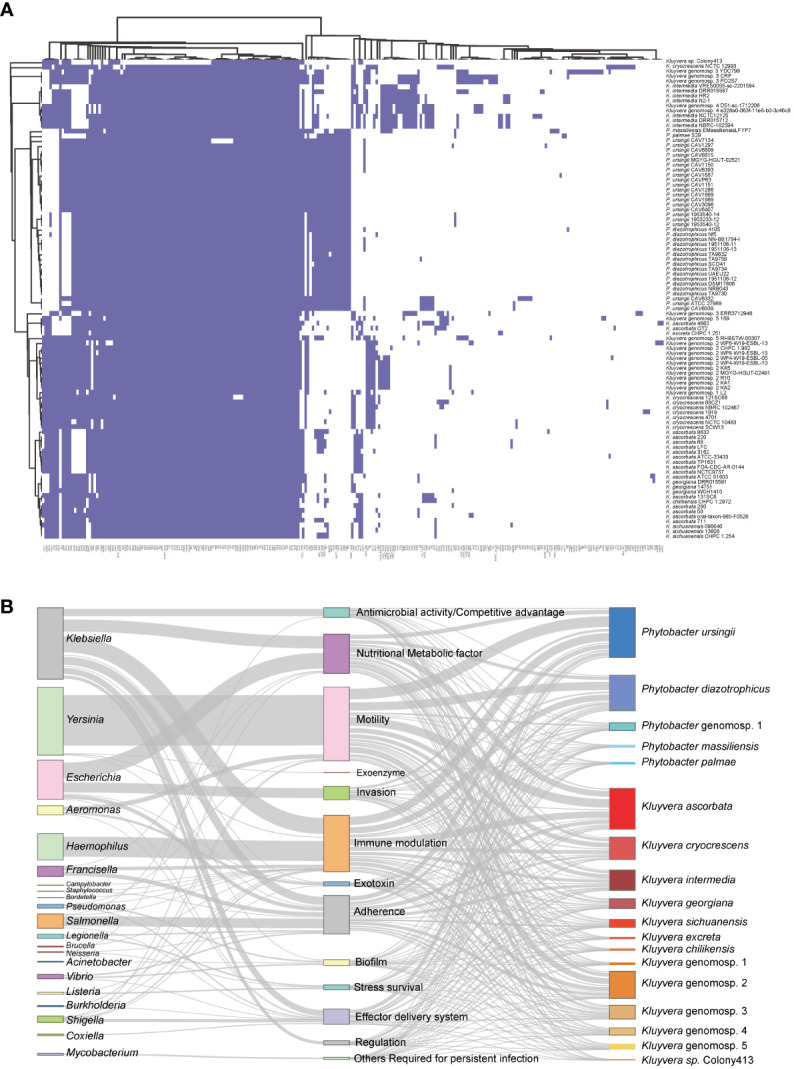
Pan virulence-related genes analysis of *Kluyvera* and *Phytobacter* genus. **(A)** The presence or absence of virulence related genes. **(B)** Virulence-related genes flow analysis. According to the BLASTp results derived from the VFDB database, virulence-related genes in *Kluyvera* and *Phytobacter* were linked to homologous genes in other species. The left column represented species share homologous virulence genes with *Kluyvera* and *Phytobacter*. The middle column represented different categories of virulence factors. The right column represented different species of *Kluyvera* and *Phytobacter*.

In total, 238 homologs of virulence-related genes were found to be present across the 97 strains, indicating a high level of divergence (**Figure 4A**). On average, each strain contained approximately 115 virulence-related genes. Among these strains, 23 virulence-related genes were shared, including *acrA*, *acrB*, *algU*, *cheA*, *flhA*, *flhB*, *flhC*, *flhD*, *fliA*, *luxS*, *rcsB*, *tufa*, *galU*, *rfaD*, *rfaE*, *rfaF*, *rpe*, *gmd*, *wza*, *gnd*, *kdsA*, *motA*, and *motB*. The genes *acrA* and *acrB*, categorized as *Klebsiella* antimicrobial activity/competitive advantage virulence factors, were involved in resistance against host-derived antimicrobial peptides (Padilla et al., 2010). The gene *algU* was a member of *Pseudomonas aeruginosa* alginate (mucoid exopolysaccharide) synthetic gene cluster, and was related with biofilm formation (Stapper et al., 2004; Nivens et al., 2001). The autoinducer-2 (AI-2) signaling molecule related gene *luxS* was presented in all 97 strains. Additionally, the gene *tufA*, which was homologous to *Francisella tularensis* elongation factor-Tu (EF-Tu), was identified as a core virulence gene in the 97 strains.

To explore the differences in pathogenicity, we further analyzed genus-specific and species-specific virulence factors. It was evident that the profiles of virulence genes did not conform to specific species, indicating their substantial diversity. Among the 60 *Kluyvera* strains, a total of 215 homologs of virulence-related genes were detected, with 37 genes belonging to the *Kluyvera* core genome. This suggests the conservative and necessary roles of these genes in *Kluyvera* pathogenesis. Among the 37 *Phytobacter* strains, 136 homologs of virulence-related genes were detected, with 72 of them belonging to the *Phytobacter* core genome. The *Kluyvera*-specific core virulence gene has not been identified thus far. However, the genes *clbS*, *csgA-C*, *hsiB1_vipA*, and *hsiC1_vipB* were found to be unique core virulence-related genes in *Phytobacter*.

We further analyzed the virulence flow to provide a comprehensive view of virulence profile. The 238 virulence-related genes mentioned above, which were of 21 different species, were divided into 13 virulence factors (**Figure 4B**). Certain genus shares fixed virulence genes with *Kluyvera* and *Phytobacter*. *Escherichia coli*-derived homologous virulence genes were the most numerous. The term of antimicrobial activity/competitive advantage was most similar to the virulence genes of the two genera (*Klebsiella*, *Neisseria*), of which *Klebsiella* accounted for 99.56% (448/450). The homologous genes of nutritional/metabolic factor were mainly derived from two genera (*Klebsiella*: 749/1847, 40.55%, *Escherichia*: 957/1847, 51.81%). The virulence factors of immune modulation had three larger sources, namely *Haemophilus influenzae* (46.80%), *Klebsiella pneumoniae* (37.5%), *Francisella tularensis* (8.1%). Remarkably, the virulence genes associated with invasion were derived only from *Escherichia*. *H. influenzae* shares only one virulence factor (immune modulation). The virulence genes homologous from *Salmonella* mainly belonged to the adherence, accounting for 32.94% (588/1785). The regulation related virulence genes were only homologous to *Klebsiella*. The flagella related genes mainly belonged to *Yersinia enterocolitica*, accounting for 90.0% (3062/3401). The exoenzyme related genes homologous to the *Yersinia pestis* were only found in the *Phytobacter palmae* strain.

### Antibiotic resistance genes (ARGs) landscape in *Kluyvera* and *Phytobacter*

**Figure 5:**
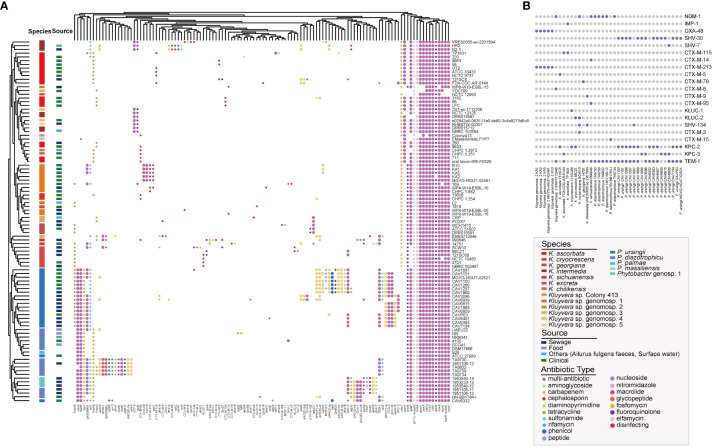
Antibiotic resistance genes (ARGs) landscape of *Kluyvera* and *Phytobacter*. **(A)** Absence and present of ARGs in *Kluyvera* and *Phytobacter* genus. **(B)** Coexistence patterns of ESBLs, *bla*_NDM_, *bla*_KPC_, and *bla*_IMP_ in *Kluyvera* and *Phytobacter* genus.

In this study, we conducted the antibiotic resistance analysis of selected strains using both genomic and phenotypic approaches. A total of 120 ARGs were identified, associated with resistance to 27 different antimicrobial drugs (**Figure 5A**). Two antibiotic resistance ontology (ARO), H-NS and CRP, were identified in the core genome of the two genera, which represented common resistance mechanisms of the resistance-nodulation-cell division (RND) antibiotic efflux pump. Besides, the RND antibiotic efflux-related ARG *baeR* was identified as a *Phytobacter*-specific resistance gene, mediating resistance to multiple drugs. Many strains exhibited the potential to be multidrug-resistant, as they carried three or more classes of antimicrobial resistance genes. Extended-spectrum β-lactamases (ESBLs) genes were detected, including *bla*_OXA-1_, *bla*_OXA-2_, *bla*_SHV-1_, *bla*_SHV-30_, *bla*_SHV-7_, *bla*_CTX-M-3_, *bla*_CTX-M-5_, *bla*_CTX-M-8_, *bla*_CTX-M-9_, *bla*_CTX-M-13_, *bla*_CTX-M-14_, *bla*_CTX-M-37_, *bla*_CTX-M-40_, *bla*_CTX-M-65_, *bla*_CTX-M-78_, *bla*_CTX-M-95_, *bla*_CTX-M-115_, *bla*_CTX-M-124-like_, *bla*_CTX-M-152_, *bla*_CTX-M-185_, *bla*_CTX-M-213_, *bla*_KLUC-1_, *bla*_KLUC-2_, *bla*_KLUC-5_, *bla*_SHV-134_, *bla*_CTX-M-15_, and *bla*_TEM-1_. Co-existence of ESBLs were identified in 25 genomes, with 18 of them belonging to *Phytobacter* strains. Notably, the presence of ESBLs and carbapenem resistance genes (*bla*_KPC_, *bla*_NDM_, *bla*_IMP_, *bla*_OXA-48_) were simultaneously detected in the genomes of 38 bacterial strains (**Figure 5B**). Twenty-one strains contained *bla*_KPC_. The co-existence of antibiotic resistance genes was more frequently observed in strains isolated from sewage samples.

## Discussion

*Kluyvera* is part of the normal microbiota of the human digestive tract (Sarria et al., 2001). But in recent years, it has been increasingly implicated in infections, even fatal cases, highlighting its emerging opportunistic nature and underestimated pathogenicity (Erbin et al., 2020). At present, *Kluyvera* comprises five recognized species and five genomospecies. In our research, we further newly complemented the members of the five genomospecies based on whole genome sequences in public databases. Previous work found that the identification of *Kluyvera* and *Phytobacter* may be problematic (Rodríguez et al., 2021; Smits et al., 2022). Classification and understanding of the genus *Phytobacter* have undergone a complex and lengthy process (Smits et al., 2022). Until year 2018, the strains associated with a multistate outbreak in Brazil were officially included in the genus *Phytobacter* (Pillonetto et al., 2018b). The genus *Phytobacter* is now recognized as an important clinically relevant member of the *Enterobacteriaceae*. It can be found in various clinical samples, such as blood, sputum, the digestive tract, and bile (Smits et al., 2022). Currently, *Phytobacter* consists of four species, *P. diazotrophicus*, *P. ursingii*, *P. palmae*, and *P. massiliensis*. To address the issue of uncertain identification and potential clinical relevance of both genera, we performed a comparative genomic analysis, focusing on taxonomy, evolution, pathogenicity, and drug resistance.

In this work, we collected all *Kluyvera* and *Phytobacter* sequences available in public databases, along with four strains isolated in this study. Using ANI (Richter and Rosselló-Móra, 2009) and *is*DDH (Meier-Kolthoff et al., 2013), we provided reliable species designations of 97 strains. Surprisingly, 41.24% of these strains were incorrectly labeled. Also, we defined a new *Phytobacter* genomospecies, namely *Phytobacter* genomospecies-1, which includes three strains (1953233-12, 1953540-12, and 1953540-14) that require phenotypic confirmation.

We calculated intergenomic distances using the GBDP algorithm (Meier-Kolthoff et al., 2013) and found that under the distance threshold of 0.15875, the best clustering decision yielded results consistent with previous identification. The pan-genome analysis of the 97 strains revealed that both *Phytobacter* and *Kluyvera* exhibit great genome plasticity and inclusiveness, likely related to their diverse ecological niches. The clustering pattern based on the core genes SNPs aligned closely with those based on whole-genome sequences, showing two distinct clusters. The long branch length of the accessory genome-based phylogenetic tree indicated a greater genetic distance compared to that of the core genome-based tree, suggesting that strains of the same species may undergo more diversification in their accessory genes due to survival pressure. Further PCoA of two genera, we identified 11 differential core genes specific to *Kluyvera* (*pelX*, *mdtL*, *bglC*, *pcak-1*, *uhpB*, *ddpA-2*, *pdxY*, *oppD-1*, *cptA*, *yidZ*, *csbX*), which could be served as potential identification targets at the genus level. Evolutionary analyses revealed the origins of the two genera and the timing of species divergence, with results suggesting that *Kluyvera* evolved at a faster rate.

By analyzing the pan virulence-related genes, we observed significant variation and high diversity among the strains, implying different levels of pathogenicity. Our study characterized the variation of virulence-related genes in the genus of *Kluyvera* and *Phytobacter*. Pan virulence-related gene analysis could help to identify virulence gene pools and explore the fundamental virulence mechanism. We identified core homologous virulence-related genes unique to *Phytobacter*, such as *clbS*, *csgA-C*, *fliS*, *hsiB1-vipA* and *hsiC1-vipB*, suggesting their role in *Phytobacter* inherent pathogenicity. Furthermore, the *Salmonella agf* genes cluster was homology to *csg* of *E. coli*, which facilitated the bacterial attachment and the villi of enterocytes (Collinson et al., 1996; White et al., 2001; White et al., 2003). The absence of the *Salmonella agf* genes cluster in *Kluyvera* strains but the presence of the complete cluster in all *Phytobacter* strains indicated that *Phytobacter* may have a stronger ability to colonize the gut. The gene *hsiB1-vipA* and *hsiC1-vipB* were homology to the gene encoding the *Pseudomonas* HSI-I (Hcp1 secretion island I) tube-sheath complex. HSI-I was highly homologous to a group of genes found in many Gram-negative proteobacteria that encoded a secretory system to play a general role in chronic *P. aeruginosa* infections (Mougous et al., 2006). Among 15 virulence related genes present in all 97 strains, the genes *acrA* and *acrB*, homologous to the genes involved in the antimicrobial activity/competitive advantage in *Klebsiella*, were responsible for mediating resistance against host-derived antimicrobial peptides (Padilla et al., 2010). Bacteria growth with alginate production was frequently as mucoidy colony, which was be consistent with culture phenotypes of the collected strains. Alginate slime layer can make it more difficult for phagocytes to ingest and kill the bacteria, suggesting a potential pathogenesis. AI-2 was produced and detected by a wide variety of bacteria and was presumed to facilitate interspecies communications (Pereira et al., 2013). For *Vibrio cholerae*, information from AI-2 mediates LuxPQ receptor complex response to allow *V. cholerae* to leave the host, re-enter the environment in large numbers and initiate a new cycle of infection (Ng and Bassler, 2009). AI-2 related gene *luxS* was presented in all 97 strains, acting as an important virulence factor. Only the strain *P. ursingii* CAV 6332 contained *allA-D*, *allR*, *allS*, which were responsible for allantion utilization in *Klebsiella*. An allantoin utilization operon has been associated with hypervirulent *K. pneumoniae* strains that cause pyogenic liver abscesses, suggesting a high pathogenesis for the *P. ursingii* strain CAV 6332.

Virulence flow analysis revealed a comprehensive view of virulence genes shared among different bacterial genera. *E. coli*-derived homologous virulence genes accounted for the highest number. The invasion related genes were only from *Escherichia*. The virulence genes belonging to the term of ‘Antimicrobial activity/Competitive advantage and Regulation’ shared the highest number with *Klebsiella*. The accessory virulence-related genes may confer an evolutionary advantage under certain environmental conditions for the strains. Remarkably, the component *chuA* of *Enterohemorrhagic E. coli* (EHEC) gene cluster (related genes: *chuA*, *chuS*, *chuT*, *chuU*, *chuW*, *chuX*, *chuY*), responsible for iron uptake, were present in all strains of *K. ascorbata*, *K. intermedia*, *Kluyvera* genomospecies-4, and *Kluyvera* genomospecies-1. And *K. ascorbata* was the species most frequently isolated in clinical specimens (Sarria et al., 2001). The gene *chuA* showed high homology to *shuA* gene of *S. dysenteriae* type 1, appearing to be widely distributed among pathogenic *E. coli* strains (Torres and Payne, 1997). Other *Kluyvera* species except above contained the remaining components of *Chu* gene cluster but not *chuA*. Therefore, *chuA* was considered as an important potential virulence gene in the pathogenic process. This result also suggested that *K. intermedia*, *Kluyvera* genomospecies-1, and *Kluyvera* genomospecies-4 were potential pathogenic species. The *chuA* gene had not been found in *Phytobacter* genomes. However, limited by the number of strains, it remains challenge to link strains virulence to the molecular and cellular processes that explain the disease in the affected host.

Antibiotic resistance is a major challenge to global public health (Laxminarayan et al., 2013). Extended-spectrum β-lactamases (ESBLs) are enzymes that hydrolyze penicillin, cephalosporins and aztreonam, leading to treatment failure (Paterson and Bonomo, 2005). They can be inhibited by enzyme inhibitors, but were sensitive to cephamycin and carbapenem antibiotics. ESBLs include TEM, SHV, CTX-M, VEB, and GES enzymes, in which CTX-M family harbored the highest number of variants (Peirano and Pitout, 2019). At present, more than 200 genotypes have been discovered, distributing in at least five subgroups (CTX-M-1, CTX-M-2, CTX-M-8, CTX-M-9, KLUC). *Kluyvera* chromosomally encoded *bla*_CTX-Ms_ was found to be the progenitors of CTX-M resistance genes, for example, CTX-M-2 genotypes were from *K. ascorbata*, KLUC were from *K. cryocrescens* and CTX-M-8 were from *K. georgiana* (Cantón et al., 2012). In this study, we identified ESBL-producing strains carrying multiple resistance genes, including various variants of *bla*_CTX-Ms_, which were the most prevalent ESBLs. Along with the changes in *Kluyvera* taxonomy, the contribution of each species to the recruitment and dissemination of their β-lactamases should be re-evaluated.

The efflux pump was to pump out the antibacterial drugs that enter the bacterial cells, thereby reducing the effective drug concentration in the bacterial cells and causing drug resistance (Li et al., 2015; Lucaßen et al., 2021). In our study, efflux pump-related genes, such as H-NS and CRP, were observed in the core genome of both genera, indicating a common resistance mechanism of resistance-nodulation-cell division (RND) antibiotic efflux pump. The bacterial efflux pump and the low permeability of the outer membrane acted synergistically to protect the bacteria from drug damage and lead to an increase in the accumulation of antimicrobial drugs in the human body (Zwama and Nishino, 2021). This will undoubtedly put stronger antibiotic selection pressure on bacteria in the human body, prompting bacteria to become more resistant.

The co-existence of two or more β-lactamases in the single strain is possibility becoming a common bacterial strategy to enhance antibiotic resistance. In our study, each strain carried multiple resistance genes, suggesting that they had the higher opportunity to become more resistant. The co-existence of ESBLs happened in the 25 strains, of which 18 were *Phytobacter* strains. Since *Phytobacter* strains were usually detected in the environment, it explained the extraordinary dispersion trend of ESBLs genes. The five strains in *Phytobacter* still contained *mcr-9.1* under the condition of containing several ESBLs resistance genes, all of which were derived from clinical samples. Carbapenems were generally considered the most effective option for infections caused by ESBLs-producing enterohepatic bacteria, but were hydrolyzed by carbapenemase (Li Y. et al., 2019). Importantly, we found co-existence of ESBLs and carbapenem resistance genes (*bla*_KPC_, *bla*_NDM_, *bla*_IMP_, *bla*_OXA-48_) in 38 strains, particularly in strains isolated from sewage. This suggests the potential development of extensively drug-resistant or even fully drug-resistant strains. Permanent selective force existing in complex environments like sewage drives diversification of the resistance mechanisms and reinforce horizontal transfer and acquisition of resistance genes. Due to the limited number of strains, the drug resistance phenotype of the strains cannot be verified. Although harboring ESBLs, two *Kluyvera* novel species identified in this study only resistant to azithromycin, a macrolide antibiotic, and sensitive to carbapenem antibiotic tested. The specific mechanism deserves further exploration. As important resistance genes reservoir, the two genera potentially become new, emerging threat as a multidrug-resistant microorganism to clinical treatment.

## Conclusion

The current studies on the two genera, *Kluyvera* and *Phytobacter*, are limited. However, this study aims to provide a comprehensive understanding of the clinical relevance and validate the species status of these two genera through whole-genome analysis. Here, we provided a better understanding of the differences between closely related species and identified previously unrecognized species. This study explores the origin and divergence time of species through evolutionary analysis. By utilizing the population structure of *Kluyvera* and *Phytobacter*, we conducted a genome-wide screening of virulence gene and resistance gene profiles. These results shed light on the divergence of virulence gene profiles and resistance gene spectra within these genera. Additionally, based on the identification of differential virulence genes, potential pathogenic mechanisms and molecular biological targets for further exploration have been proposed. The importance of *Kluyvera* and *Phytobacter* as emerging causes of human disease has been emphasized in this study. Moreover, the study highlights the potential of these two genera as multidrug-resistant microorganisms, posing a threat to clinical treatment.

## Data availability statement

The datasets presented in this study can be found in online repositories. The names of the repository/repositories and accession number(s) can be found in the article/**Supplementary Material**.

## Author contributions

ZH: Conceptualization, Data curation, Formal analysis, Software, Visualization, Writing – original draft. GZ: Data curation, Investigation, Methodology, Writing – original draft. ZZ: Conceptualization, Data curation, Investigation, Methodology, Writing – original draft. XL: Formal analysis, Investigation, Resources, Writing – original draft. FC: Formal analysis, Investigation, Methodology, Writing – original draft. LZ: Formal analysis, Investigation, Resources, Writing – original draft. DW: Investigation, Resources, Supervision, Validation, Writing – review & editing. KY: Conceptualization, Formal analysis, Investigation, Project administration, Resources, Supervision, Validation, Visualization, Writing – review & editing. JL: Conceptualization, Funding acquisition, Investigation, Project administration, Resources, Supervision, Validation, Visualization, Writing – review & editing.
